# Greater Protective Potent of s-Methyl Cysteine and Syringic Acid Combination for NGF-differentiated PC12 Cells against Kainic acid-induced Injury

**DOI:** 10.7150/ijms.35083

**Published:** 2019-08-06

**Authors:** I-ching Chou, Mei-chin Mong, Chih-lung Lin, Mei-chin Yin

**Affiliations:** 1 Division of Pediatric Neurology, China Medical University Hospital, Taichung City, Taiwan; 2 Department of Food Nutrition and Health Biotechnology, Asia University, Taichung City, Taiwan; 3 Department of Neurosurgery, Asia University Hospital, Taichung City, Taiwan; 4 Department of Medical Research, China Medical University Hospital, China Medical University, Taichung City, Taiwan

**Keywords:** seizure, s-methyl cysteine, syringic acid, glutamine, calcium release, p38MAPK

## Abstract

**Objective:** The effects of pre-treatments from s-methyl cysteine (SMC) alone, syringic acid (SA) alone and SMC plus SA against kainic acid (KA) induced injury in nerve growth factor (NGF) differentiated PC12 cells were investigated.

**Methods:** NGF-differentiated PC12 cells were treated with 1 μM SMC, 1 μM SA or 0.5 μM SMC plus 0.5 μM SA for 2 days. Subsequently, cells were further treated by 150 μM KA.

**Results:** KA suppressed Bcl-2 mRNA expression, enhanced Bax mRNA expression and casued cell death. SMC was greater than SA, and similar as SMC+SA in increasing Bcl-2 mRNA expression. SMC+SA led to greater increase in mitochondrial membrane potential and cell survival than SMC or SA alone. SMC+SA resulted in more reduction in reactive oxygen species and tumor necrosis factor-alpha generation, more increase in glutathione content and glutathione reductase activity than SMC or SA alone. KA up-regulated protein expression of nuclear factor kappa B (NF-κB) p65 and phosphorylated p38 (p-p38). SMC or SA pre-treatments alone limited protein expression of both factors. SMC+SA resulted in more suppression in NF-κB p65 and p-p38 expression. KA decreased glutamine level, increased glutamate level and stimulated calcium release. SMC pre-treatments alone reversed these alterations. SMC alone elevated glutamine synthetase (GS) activity and mRNA expression. SMC+SA led to greater GS activity and mRNA expression than SMC pre-treatments alone.

**Conclusion:** These findings suggested that this combination, SMC+SA, might provide greater protective potent for neuronal cells.

## Introduction

s-Methyl cysteine (SMC) is a cysteine-containing compound naturally presented in many edible *Allium* plants like garlic and onion. It is reported that dietary intake of SMC, a hydrophilic agent, could enhance the anti-oxidative defense in organs through increasing glutathione (GSH) content 1. Furthermore, animal studies revealed that SMC displayed multiple protective functions upon brain against neuropsychiatric disorders and Parkinson's disease 2, 3. Syringic acid (SA) is a phenolic acid naturally occurred in several medicinal plants such as *Morus nigra* and *Daphne gnidioides* 4, 5. The protective effects of SA, a lipophilic compound, in mice brain and cultured hippocampal neuronal cells have been observed 5, 6. Those authors indicated that SA could ameliorate oxidative injury in brain and neuronal cells. So far, less attention was paid to the individual effects of SMC or SA against seizure. In addition, it also remains unknown whether the combination of SMC plus SA could exhibit additive or greater potent against seizure. The idea regarding the combined effects of SMC plus SA is from the study of Kumral et al. 7, in which a hydrophilic agent, carnosine, plus a lipophilic agent, vitamin E, provided more significantly anti-oxidative and anti-inflammatory activities in heart and liver of rats against doxorubicin than carnosine or vitamin E treatments alone. It is possible that the combination of two compounds with different biochemical properties may be more potential in disease prevention or alleviation via different action modes.

The pathological characteristics of seizure are involuntary shaking of partial or entire body 8. The etiopathogenesis of seizure is ascribed to inflammatory and oxidative stresses in the area of hippocampus 9, 10. The activation of signalling pathways such as p38 mitogen-activated protein kinase (MAPK) and nuclear factor kappa B (NF-κB) results in massive production of down-stream inflammatory and oxidant factors including interleukin (IL)-1beta, IL-6, tumor necrosis factor (TNF)-alpha and reactive oxygen species (ROS), and these elements consequently facilitate the progression of seizure 11, 12. On the other hand, glutamate excitotoxicity plays another crucial role responsible for seizure induction because increased extracellular glutamate in hyper-excitable areas of brain stimulates neuronal excitability 13, 14. Glutamine synthetase (GS) benefits glutamate clearance through metabolizing glutamate to glutamine 15. If SMC and SA, alone or combined, could suppress p38MAPK and NF-κB pathways, increase GS activity and mitigate glutamate toxicity, they may ameliorate seizure severity. Kainic acid (KA), a glutamate related compound, could release calcium ions to impair nerve impulse transmission, which induces neuronal membrane depolarization and causes neuronal excitability 16. KA-induced seizure in rodents has been widely used as an experimental lesional seizure model for the associated pathological, preventive and therapeutic researches because KA causes focal hippocampal lesion 17, 18.

In our present study, the effects of pre-treatments from SMC alone, SA alone and SMC plus SA against KA induced multiple injuries in nerve growth factor (NGF) differentiated PC12 cells were investigated. The impact of these treatments upon viability, plasma membrane stability, mRNA and protein expression of associated factors, calcium release and glutamate clearance of these cells was evaluated. These results could elucidate the possibility of using SMC and SA, alone or combined, to prevent or alleviate seizure.

## Materials and methods

### Materials

SMC (99.5%), SA (98%), NGF (99%) and KA (99.5%) were purchased from Wako Chem. Co. (Tokyo, Japan). Antibodies and materials for cell culture were obtained from Sigma Chem. Co. (St. Louis, MO, USA).

### Cell culture and treatments

PC12 cells were routinely cultured in Dulbecco's modified Eagle's medium (DMEM) and maintained at 37°C under 95% air and 5% CO_2_. NGF at 50 ng/ml was used to treat PC12 cells, and followed by incubating for 5 days at 37°C for differentiation. DMEM was refreshed every 3 days, and cells were sub-cultured every 7 days. Cells were further planted in 96 well plates after washing twice with serum-free DMEM. Cell number at 10^5^/ml, adjusted by phosphate-buffered saline (PBS, pH 7.2), was used for experiments. SA at 200 mg was suspended in 2 ml 0.8% methyl cellulose, and diluted with DMEM. SMC or KA was dissolved in PBS. Cells were treated with 1 μM SMC, 1 μM SA or 0.5 μM SMC plus 0.5 μM SA for 2 days at 37°C. Subsequently, cells were further treated with 150 μM KA. Cells without test compounds and KA were normal groups. Cells without test compounds, but with KA were control groups (KA groups).

### Measurement of cell viability and plasma membrane damage

One day after KA treatment, 3-(4,5-dimethylthiazol-2-yl)-2,5-diphenyltetrazolium bromide (MTT) at 0.25 mg/ml was added into cell suspension. After incubating for 3 hr at 37°C, the absorbance at 570 nm was read to measure MTT formazan product by a microplate reader (Bio-Rad, Hercules, CA, USA). Cell viability was expressed as a percentage of normal groups. Plasma membrane damage was determined by analyzing the activity (U/l) of lactate dehydrogenase (LDH). After centrifuging, 50 μl of cell supernatant was collected. LDH activity was assayed by a commercial kit (Sigma Chem. Co., St. Louis, MO, USA).

### Determination of mitochondrial membrane potential (MMP) and DNA fragmentation

MMP was measured by using Rh123, a fluorescent dye. Cell suspension was mixed with 100 μg/l of Rh123 for 30 min at 37°C. After washing twice with PBS, the mean fluorescence intensity (MFI) was measured by a FC500 flow cytometry (Beckman Coulter, Fullerton, CA, USA). MFI was used as an indicator of MMP. DNA fragmentation was quantified by cell death detection kit (Roche Molecular Biochemicals, Mannheim, Germany). Cells were suspended in lysis buffer, and followed by incubating for 30 min at 25°C. After centrifuging for 10 min at 250 xg, 20 μl of cell supernatant was used to react with 80 μl of immunoreagents. After incubating for 2 hr at 25°C and washing twice with PBS, reactive substrate was added and further incubated for 15 min. The absorbance at 405 nm and 490 nm was read by a Bio-Rad microplate reader. Enrichment factor, (absorbance of the sample)÷(absorbance of the control), was shown to indicate DNA fragmentation.

### Measurement of oxidative and inflammatory factors

Protein content of 100 μl cell homogenate was quantified by an assay kit (Pierce, Rockford, IL, USA). GSH level was measured by a kit purchased from EMD Biosciences Inc. (San Diego, CA, USA). The activities of glutathione reductase (GR) or peroxidase (GPX) were determined by assay kits (OxisResearch Co., Portland, OR, USA) according to manufacturers' instructions. 2',7'-Dichlorofluorescein diacetate (DCFH-DA) was used to detect ROS level. DCFH-DA at 2 mg/ml was reacted with 100 μl cell homogenate, and followed by incubating for 30 min at 37°C. Fluorescence value was read by a fluorescence plate reader (Molecular Devices, Sunnyvale, CA, USA) with emission and excitation wavelengths at 525 nm and 488 nm, respectively. Result was shown as relative fluorescence unit (RFU)/mg protein. Cyclooxygenase (COX)-2 activity, IL-6, TNF-alpha and prostaglandin E (PGE)_2_ levels were assayed by kits purchased from Cayman Chem. Co. (Ann Arbor, MI, USA) according to manufacturer's instructions.

### Assay for NF-κB p50/65 binding activity

Nuclear protein of cells was extracted and isolated according to the method of Schilling et al. 19. Nuclear protein extract at 10 μg was applied for detecting NF-κB p50/65 binding activity via a commercial kit obtained from Chemicon International Co. (Temecula, CA, USA). A primary NF-κB p50/p65 antibody, 3, 3′, 5, 5′-tetramethylbenzidine, was mixed with nuclear protein. Sample was incubated for 1 hr at 25°C and washed twice with PBS. Then, horseradish peroxidase-conjugated antibody was added, and followed by incubating for another 1 hr. The absorbance at 450 nm was read by a Bio-Rad microtiter plate reader (Model 550, Hercules, CA, USA). Result was expressed as folds of normal groups.

### Protein expression of NF-κB p65 and p38MAPK

Protein concentration of cell homogenate was analyzed by assay reagents obtained from Bio-Rad Laboratories Inc. (Hercules, CA, USA). Sample with 30 μg protein was applied to determine protein expression of NF-κB p65 and phosphorylated p38 (p-p38) by commercial ELISA kits (Abcam Co., Cambridge, MA, USA) according to the instructions of manufacturers.

### Analyses of glutamate, glutamine and GS activity

For analyzing glutamate or glutamine content, cell homogenate was respectively mixed with sodium citrate buffer or lithium citrate buffer. After centrifuging, the level of glutamate or glutamine in supernatant was measured by an amino acid analyzer (L-8800, Hitachi, Tokyo, Japan). The peak of glutamate or glutamine was first identified by its retention time through comparing with its external standard, and the concentration (ng/mg protein) was quantified according to the peak height. GS activity was measured according to the method of Castegna et al. 20. Protein content of cell homogenate was determined, 200 μg protein was used for analyzing GS activity. Cell protein was mixed with phosphoenolpyruvate, freshly prepared NADH, pyruvate kinase, and lactate dehydrogenase to 100 μl final volume. Then, 900 μl of reaction cocktail containing imidazole, glutamate, ATP, MgCl_2_, KCl, and NH_4_Cl (pH 7.1) was added. The variation of NADH level was determined by monitoring the absorbance at 340 nm for 10 min. Result was shown in μmol/min/mg protein.

### Quantification of calcium level

The level of intracellular Ca^2+^ was measured by a Ca^2+^-sensitive dye, Fura-2AM 21. Fura-2AM at 5 mmol/l was mixed with 0.1% dimethyl sulfoxide and 1% bovine serum albumin. Cell homogenate was added, and followed by incubating for 30 min at 37°C in dark condition. Fluorescence variation was read by a spectrofluorimeter (Model RF-5000, Shimadzu, Kyoto, Japan) with wavelengths at 340 and 380 nm for excitation, and wavelength at 510 nm for emission. Calcium level (nM) was calculated by the equation: *K*d×[(*R*-*R*min)÷(*R*max-*R*)]×FD÷FS. *K*d was 224. *R* was the value of 340/380. *R*min or *R*max was determined by treating cells respectively with ethylene glycol tetra-acetic acid or triton X-100. FD was the fluorescence value of Ca^2+^-free form obtained at 380 nm. FS was the fluorescence value of Ca^2+^-bound form obtained at 340 nm.

### Real-time polymerase chain reaction (RT-PCR) for mRNA expression

Total mRNA was isolated from cells, and RNA concentration was quantified by reading the absorbance at 260 nm. Subsequently, 5 μg RNA was used to synthesize cDNA by a commercial cDNA synthesis kit (Legene Biosciences, San Diego, CA, USA). cDNA was applied for RT-PCR process. The used oligonucleotide primers included Bcl-2, forward, 5'-GTG GAT GAC TGA GTA CCT GAA C-3', reverse, 5'-GAG ACA GCC AGG AGA AAT CAA-3'; Bax, forward, 5'-GCT GAT GGC AAC TTC AAC TG-3', reverse, 5'-ATC AGC TCG GGC ACT TTA G-3'; GS, forward, 5'**-**CCA CTG TCC CTG GGC TTA GTT TA-3', reverse, 5'-AGT GAC ATG CTA GTC CCA CCA A-3'; glyceraldehyde-3-phosphate dehydrogenase (GAPDH), forward, 5'-AGA GGC AGG GAT GTT CTG-3', reverse, 5'-GAC TCA TGA CCA CAG TCC ATG C-3'. cDNA amplification condition was 95ºC for 3 min, 95ºC for 10s and 56ºC for 30s. Forty cycles were run for Bcl-2, Bax and GS, and 28 cycles were run for GAPDH. The produced fluorescence was determined by a Taqman system with real-time sequence detection, and mRNA level was shown as a percentage of normal groups.

### Statistical analyses

The effect of each treatment was obtained from 8 different preparations (n=8). Data were shown as mean ± standard deviation (SD). Statistical analyses were treated by one-way analysis of variance, and processed by SAS (SAS Institute, Cary, NC, USA). Dunnett's* t*-test was used for Post-hoc comparison. Difference was considered as significant when *p* value was < 0.05.

## Results

### SMC and SA improved cell viability and plasma membrane damage

**Figure 1:**
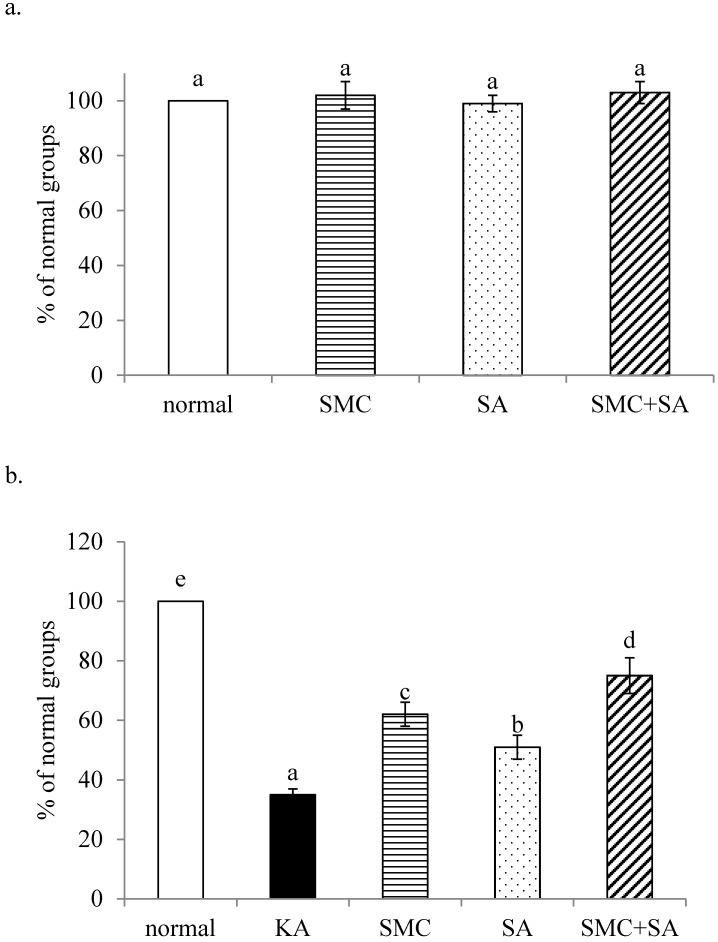
Effects of SMC and/or SA upon cell viability. NGF-differentiated PC12 cells were pre-treated with 1 μM SMC, 1 μM SA or 0.5 μM SMC+0.5 μM SA, and without (a) or with (b) KA treatment. Normal group had no SMC, SA, or KA. Data are mean ± SD (n=8). ^a-e^Values among bars without a common letter differ, *p*<0.05.

**Figure 2:**
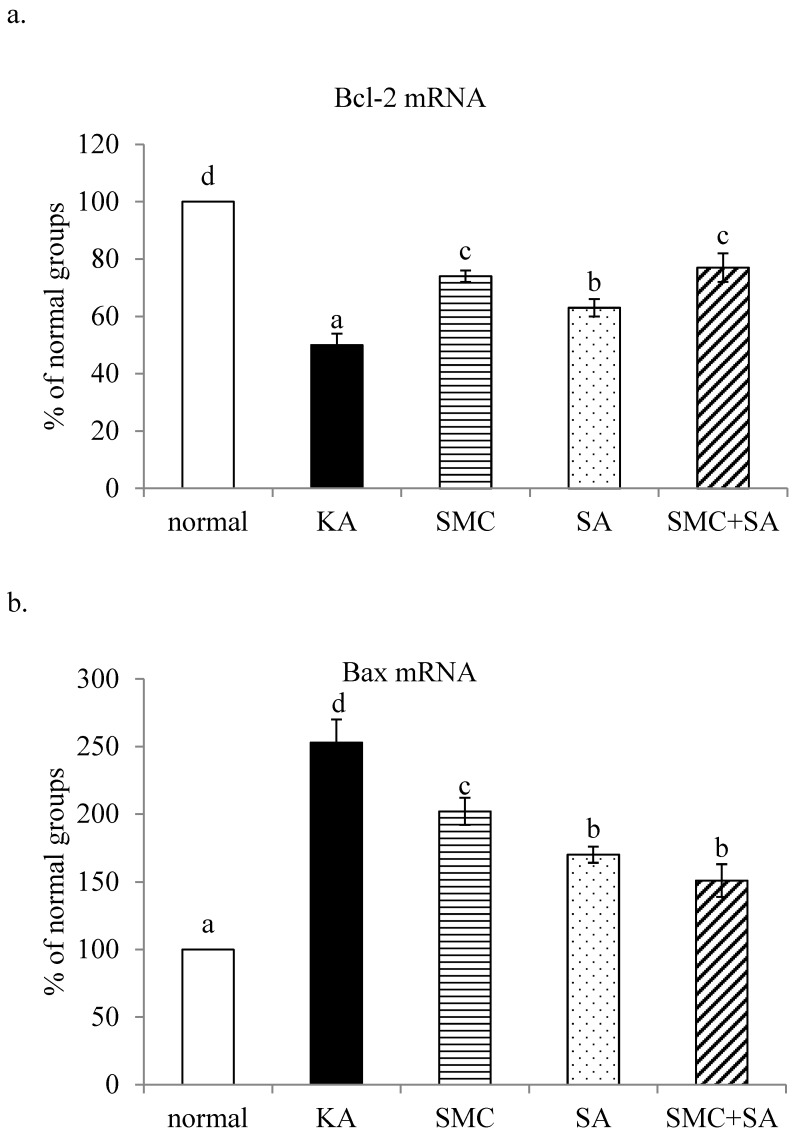
Effects of SMC and/or SA upon mRNA expression of Bcl-2 (a) and mRNA expression of Bax (b). NGF-differentiated PC12 cells were pre-treated with 1 μM SMC, 1 μM SA or 0.5 μM SMC+0.5 μM SA, and followed by using KA to induce cell injury. Normal group had no SMC, SA, or KA. Data are mean ± SD (n=8). ^a-e^Values among bars without a common letter differ, *p*<0.05.

Without KA treatment, SMC and/or SA pre-treatments did not affect cell viability (Figure 1a, *p*>0.05). As shown in Figure 1b, KA induced cell death, and SMC or SA pre-treatments alone increased cell viability (*p*<0.05). SMC+SA pre-treatments led to greater cell survival than SMC or SA alone (*p*<0.05). KA suppressed Bcl-2 mRNA expression and enhanced Bax mRNA expression (Figure 2a and 2b, *p*<0.05). SMC was greater than SA, and similar as SMC+SA in increasing Bcl-2 mRNA expression (2a, *p*<0.05). SA was greater than SMC, and similar as SMC+SA in limiting Bax mRNA expression (2b, *p*<0.05). SMC or SA pre-treatments decreased LDH activity, raised MMP and lowered DNA fragmentation (Table 1, *p*<0.05). SMC was more effective than SA in decreasing DNA fragmentation (*p*<0.05). SMC+SA caused greater reduction in LDH activity, and greater increase in MMP than SMC or SA treatments alone (*p*<0.05).

### SMC and SA attenuated oxidative and inflammatory stresses

As shown in Table 2, SMC or SA pre-treatments decreased ROS production, increased GSH level, raised activity of GPX and GR (*p*<0.05). SMC was more effective than SA in changing those four examined parameters (*p*<0.05). SMC+SA resulted in greater decrease in ROS generation, greater increase in GSH content and GR activity than SMC or SA pre-treatments alone (*p*<0.05). SMC or SA pre-treatments lowered the level of IL-6, TNF-alpha and PGE_2_, as well as reduced COX-2 activity (Table 3, *p*<0.05). SA was greater than SMC in changing those inflammatory parameters (*p*<0.05). SMC+SA led to more decrease in TNF-alpha than SMC or SA pre-treatments alone (*p*<0.05).

### SMC and SA suppressed NF-κB and p38MAPK

**Figure 3:**
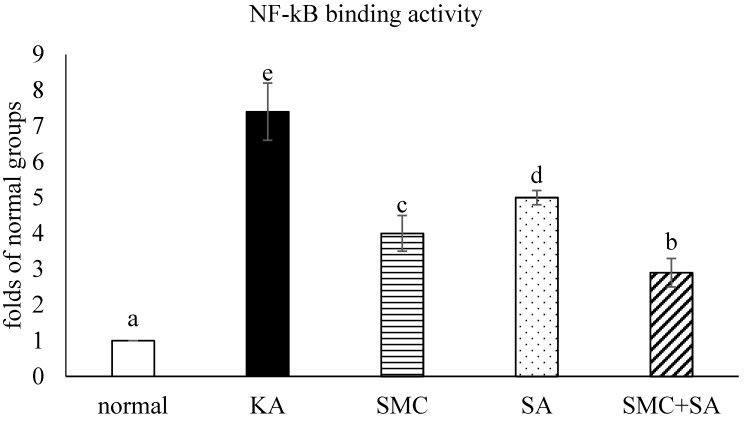
Effects of SMC and/or SA upon NF-κB p50/65 binding. NGF-differentiated PC12 cells were pre-treated with 1 μM SMC, 1 μM SA or 0.5 μM SMC+0.5 μM SA, and followed by using KA to induce cell injury. Normal group had no SMC, SA, or KA. Data are mean ± SD (n=8). ^a-e^Values among bars without a common letter differ, *p*<0.05.

**Figure 4:**
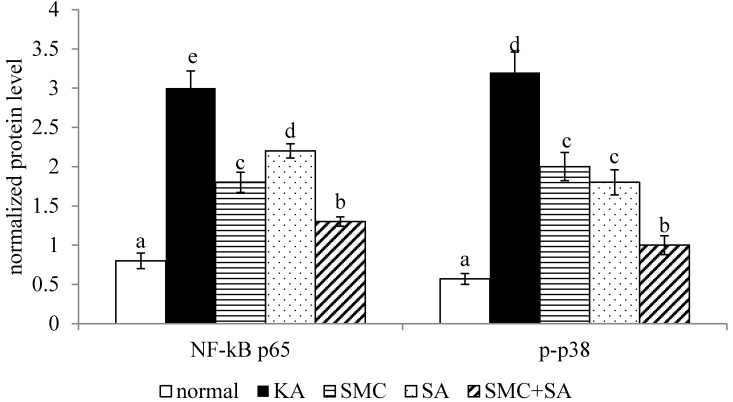
Effects of SMC and/or SA upon protein expression of NF-κB p65 and p-p38. NGF-differentiated PC12 cells were pre-treated with 1 μM SMC, 1 μM SA or 0.5 μM SMC+0.5 μM SA, and followed by using KA to induce cell injury. Normal group had no SMC, SA, or KA. Data are mean ± SD (n=8). ^a-e^Values among bars without a common letter differ, *p*<0.05.

KA increased NF-κB p50/65 binding activity (Figure 3, *p*<0.05). SMC or SA pre-treatments alone diminished NF-κB p50/65 binding activity (*p*<0.05), in which SMC was greater than SA in reducing this activity (*p*<0.05). SMC+SA led to more reduction in this activity than SMC or SA pre-treatments alone (*p*<0.05). As shown in Figure 4, KA up-regulated protein expression of NF-κB p65 and p-p38 (*p*<0.05). SMC or SA pre-treatments alone down-regulated protein expression of NF-κB p65 and p-p38 (*p*<0.05), in which SMC was greater than SA in lowering NF-κB p65 expression (*p*<0.05). SMC+SA resulted in more suppression in NF-κB p65 and p-p38 expression (*p*<0.05).

### SMC and SA altered glutamate, glutamine, GS activity and calcium release

**Figure 5:**
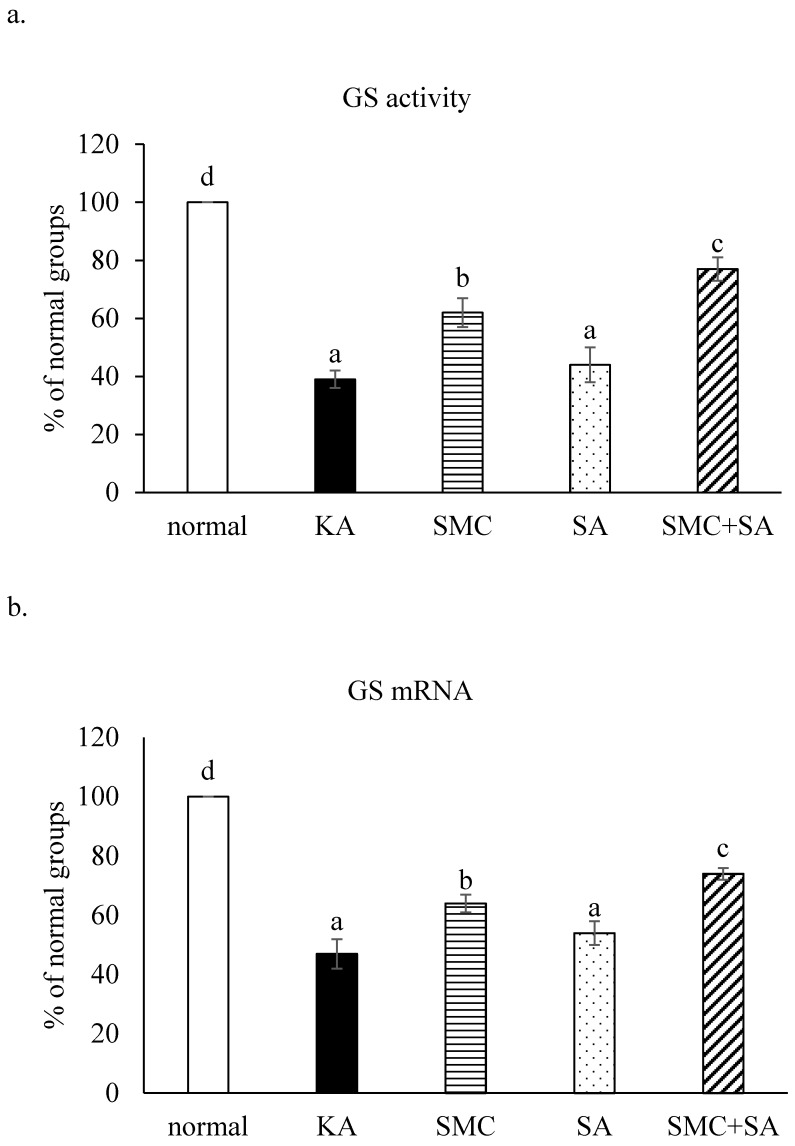
Effects of SMC and/or SA upon GS activity (a) and GS mRNA expression (b). NGF-differentiated PC12 cells were pre-treated with 1 μM SMC, 1 μM SA or 0.5 μM SMC+0.5 μM SA, and followed by using KA to induce cell injury. Normal group had no SMC, SA, or KA. Data are mean ± SD (n=8). ^a-d^Values among bars without a common letter differ, *p*<0.05.

KA decreased glutamine level and increased glutamate level, as well as stimulated calcium release (Table 4, *p*<0.05). SMC pre-treatments alone reversed these alterations (*p*<0.05). SA pre-treatments alone mildly raised glutamine level (*p*<0.05), but failed to affect glutamate level and calcium release (*p*>0.05). KA limited GS activity and GS mRNA expression (Figure 5, *p*<0.05). SMC, not SA, pre-treatments alone elevated GS activity and mRNA expression (*p*<0.05). SMC+SA led to more increase in GS activity and mRNA expression than SMC pre-treatments alone (*p*<0.05).

## Discussion

Either SMC or SA could attenuate KA induced oxidative and inflammatory stresses in NGF-differentiated PC12 cells, which contributed to enhance cell survival. However, SMC, not SA, might be a potent agent for ameliorating seizure because SMC alone could diminish calcium release and attenuate glutamate excitotoxicity via promoting GS activity and mRNA expression. Furthermore, our data revealed that SMC+SA was more effective than SMC or SA alone to alleviate seizure associated pathological factors, which were evidenced by less released calcium, greater GS activity and mRNA expression, lower expression of NF-κB p65 and p-p38, greater cell viability and plasma membrane protection. These novel findings suggested that SMC+SA might provide more protective activities against seizure.

KA up-regulated Bax mRNA expression and down-regulated Bcl-2 mRNA expression, which in turn evoked apoptotic stress in cells. Consequently, the loss of KA treated cells could be explained. We found SMC, SA or SMC+SA pre-treatments increased Bcl-2 mRNA expression and limited Bax mRNA expression, which subsequently mitigated cellular apoptotic injury. In addition, the decreased mitochondrial membrane potential and increased DNA fragmentation are the events strongly associated with cell rupture 22. Thus, the improvement from SMC and/or SA upon plasma membrane damage and DNA fragmentation elicited by KA indicated that these agents alone or combined could enhance the stability of DNA and plasma membrane. These results implied that SMC and SA might be able to penetrate into NGF-differentiated PC12 cells and benefit cell survival. SMC is a hydrophilic agent and SA is a lipophilic agent. Based on the different chemical properties, it is highly possible that these two agents cooperatively attenuated KA induced injury or toxicity in cellular aqueous and lipidic regions, respectively. Consequently, this combination, SMC+SA, provided stronger ability to stabilize cellular compartments.

Increased oxidative and inflammatory stresses due to massive production of oxidants and cytokines including ROS, IL-6 and TNF-alpha are responsible for the relapse and severity of seizure 23, 24. It is indicated that COX-2 activity is rapidly induced during seizure, which leads to the synthesis of PGE_2_, and favors the progression or deterioration of seizure 25. Our present study found that SMC treatments could increase GSH level through raising both GPX and GR activities. These results suggest that SMC enhanced anti-oxidative defense via promoting GSH homeostasis in NGF-differentiated PC12 cells. Although SA was a weaker antioxidant when compared with SMC, it displayed marked anti-inflammatory activities because its inhibitory effects upon COX-2 activity and PGE_2_ generation were stronger than SMC. Thus, the greater potent of this SMC+SA combination against seizure might be due to SMC diminishing oxidative stress and SA mitigating inflammatory injury. On the other hand, the activation of NF-κB and p38MAPK from stimulators such as KA facilitates the formation of down-stream oxidants and inflammatory mediators 26, 27. It is well known that the activation of these two signaling pathways plays crucial role in seizure development and progression 28, 29. We found that SMC+SA more significantly limited NF-κB p50/p65 binding activity, and suppressed the protein expression of NF-κB p65 and p-p38. Since these up-stream regulators have been restricted, it was reasonable to observe the lower production of ROS and inflammatory cytokines. In fact, it has been reported that SMC and SA possessed anti-oxidative and/or anti-inflammatory activities 30, 31. Our data further revealed that the combination, SMC+SA, more efficiently mediated NF-κB and p38MAPK pathways, and exhibited greater anti-oxidative and anti-inflammatory protection for NGF-differentiated PC12 cells against KA. These findings suggested that this combination might be a powerful agent against seizure.

GS is an enzyme responsible for converting glutamate to glutamine. Lower GS activity or mRNA expression due to KA treatment impairs this conversion and results in the accumulation of glutamate 32. Higher extracellular glutamate caused glutamate excitotoxicity because extra glutamates promote synaptic excitation of central nervous system 33. In addition, extra glutamates block cellular uptake for cysteine and reduce intracellular cysteine level 34, which in turn decreases available cysteine for GSH synthesis and exacerbates oxidative injury 35. Our data revealed that SMC and SMC+SA markedly restored GS activity and mRNA expression in KA treated cells, which subsequently contributed to convert glutamate to glutamine, and finally attenuated glutamate induced synaptic excitation. The lower glutamate and higher glutamine levels observed in SMC and SMC+SA groups agreed that SMC or SMC+SA were effective agents for glutamate clearance. On the other hands, the declined glutamate also improved glutamate induced oxidative stress.

Malve et al. 16 reported that massive release of Ca^2+^ in neuronal cells intercepts nerve impulse transmission, evokes neuronal excitability, and causes the recurrence of seizure. We found SMC and SMC+SA substantially lowered Ca^2+^ release induced by KA in NGF-differentiated PC12 cells. These data suggested that SMC and SMC+SA were able to attenuate seizure through restricting Ca^2+^ release in neuronal cells. Nagarkatti et al. 36 reported that excessive glutamate stimulated N-methyl-D-aspartate (NMDA) receptors that are Ca^2+^-permeable ion channels, which in turn caused the movement of Ca^2+^ from extracellular region into the cells. Since SMC and SMC+SA already decreased glutamate and maintained plasma membrane integrity, the stimulation from glutamate upon NMDA receptors was declined, which subsequently lowered the released Ca^2+^ in those groups. These results implied that SMC and SMC+SA might be potent inhibitors for NMDA receptors. SMC is an amino acid derivate, and SA is a phenolic acid presented in many edible plant foods. The application of these two natural compounds should be safe. Further *in vivo* study is necessary to verify the effects and safety of this combination against seizure.

In conclusion, the combination of s-methyl cysteine and syringic acid exhibited greater protective effects for NGF-differentiated PC12 cells against kainic acid induced apoptotic, oxidative and inflammatory injury than s-methyl cysteine or syringic acid treatments alone. These findings suggested that this combination might provide greater protective potent for neuronal cells.

## Figures and Tables

**Table 1:** Effects of SMC and/or SA upon LDH activity, MMP and DNA fragmentation. NGF-differentiated PC12 cells were pre-treated with 1 μM SMC, 1 μM SA or 0.5 μM SMC+0.5 μM SA, and followed by using KA to induce cell injury. Normal group had no SMC, SA, or KA. Data are mean ± SD (n=8). ^a-d^Values in a column without a common letter differ, *p*<0.05.

	LDH U/l	MMP % of normal groups	DNA fragmentation Folds of normal groups
Normal	37±4^a^	100^d^	1.00^a^
KA	220±18^d^	29±5^a^	2.42±0.15^d^
SMC	142±16^c^	57±8^b^	1.47±0.1^b^
SA	159±9^c^	51±6^b^	1.74±0.11^c^
SMC+SA	100±7^b^	68±4^c^	1.38±0.07^b^

**Table 2:** Effects of SMC and/or SA upon level or activity of ROS, GSH, GPX and GR. NGF-differentiated PC12 cells were pre-treated with 1 μM SMC, 1 μM SA or 0.5 μM SMC+0.5 μM SA, and followed by using KA to induce cell injury. Normal group had no SMC, SA, or KA. Data are mean ± SD (n=8). ^a-e^Values in a column without a common letter differ, *p*<0.05.

	ROS RFU/mg protein	GSH ng/mg protein	GPX U/mg protein	GR U/mg protein
Normal	0.11±0.04^a^	93±2^e^	68.2±3.4^d^	63.1±1.3^e^
KA	2.08±0.17^e^	31±4^a^	32.0±2.3^a^	30.5±0.8^a^
SMC	1.31±0.12^c^	63±5^c^	52.6±1.8^c^	45.7±1.2^c^
SA	1.68±0.13^d^	48±3^b^	43.5±2.5^b^	38.3±1.0^b^
SMC+SA	0.94±0.08^b^	74±4^d^	55.4±1.7^c^	53.6±0.7^d^

**Table 3:** Effects of SMC and/or SA upon IL-6, TNF-alpha and PGE_2_ levels, and COX-2 activity. NGF-differentiated PC12 cells were pre-treated with 1 μM SMC, 1 μM SA or 0.5 μM SMC+0.5 μM SA, and followed by using KA to induce cell injury. Normal group had no SMC, SA, or KA. Data are mean ± SD (n=8). ^a-e^Values in a column without a common letter differ, *p*<0.05.

	IL-6 pg/mg protein	TNF-alpha pg/mg protein	PGE_2_ pg/mg protein	COX-2 U/mg protein
Normal	13±3^a^	9±5^a^	135±10^a^	0.09±0.02^a^
KA	103±7^d^	110±4^e^	347±19^d^	1.48±0.08^d^
SMC	84±4^c^	90±3^d^	280±12^c^	1.16±0.1^c^
SA	73±2^b^	71±6^c^	232±8^b^	0.83±0.07^b^
SMC+SA	67±5^b^	58±4^b^	219±12^b^	0.71±0.11^b^

**Table 4:** Effects of SMC and/or SA upon level of glutamine, glutamate and calcium release. NGF-differentiated PC12 cells were pre-treated with 1 μM SMC, 1 μM SA or 0.5 μM SMC+0.5 μM SA, and followed by using KA to induce cell injury. Normal group had no SMC, SA, or KA. Data are mean ± SD (n=8). ^a-e^Values in a column without a common letter differ, *p*<0.05.

	glutamine ng/mg protein	glutamate ng/mg protein	Ca^2+^ nM
Normal	740±39^e^	172±20^a^	239±24^a^
KA	139±19^a^	1417±155^d^	1674±119^d^
SMC	435±27^c^	903±58^c^	863±42^c^
SA	218±30^b^	1258±72^d^	1490±88^d^
SMC+SA	526±34^d^	729±68^b^	685±37^b^

## Abbreviations

COX: cyclooxygenase
DCFH-DA: 2',7'-Dichlorofluorescein diacetate
DMEM: dulbecco's modified Eagle's medium
GAPDH: glyceraldehyde-3-phosphate dehydrogenase
GPX: glutathione peroxidase
GR: glutathione reductase
GS: glutamine synthetase
GSH: glutathione
IL: interleukin
KA: kainic acid
LDH: lactate dehydrogenase
MAPK: mitogen-activated protein kinase
MMP: mitochondrial membrane potential
MTT: 3-(4,5-dimethylthiazol-2-yl)-2,5-diphenyltetrazolium bromide
NF-κB: nuclear factor kappa B
NGF: nerve growth factor
NMDA: N-methyl-D-aspartate
PGE: prostaglandin E
RFU: relative fluorescence unit
ROS: reactive oxygen species
RT-PCR: real-time polymerase chain reaction
SA: syringic acid
SMC: s-methyl cysteine
TNF: tumor necrosis factor
